# Species-specific coevolution of RecA–RecN interfaces governs DNA double-strand break repair in *Escherichia coli*

**DOI:** 10.1371/journal.pgen.1012169

**Published:** 2026-05-28

**Authors:** Mizuki Inoue, Genki Akanuma, Masafumi Hayashi, Takashi Hishida

**Affiliations:** 1 Department of Molecular Biology, Graduate School of Science, Gakushuin University, Tokyo, Japan; 2 Department of Chemistry and Biological Science, Faculty of Science, Josai University, Saitama, Japan; University of Wisconsin-Madison, UNITED STATES OF AMERICA

## Abstract

DNA double-strand breaks (DSBs) threaten genome stability and cell survival but can be faithfully repaired through homologous recombination (HR). RecN, a bacterial protein closely related to the structural maintenance of chromosomes family, cooperates with RecA in HR-dependent DSB repair, yet the molecular basis and physiological relevance of their interaction remain unclear. Here, we investigated the functional interplay between RecA and RecN during DSB repair by heterologously expressing *Pseudomonas aeruginosa* RecA (paRecA) and RecN (paRecN) in *Escherichia coli.* We found that in *E. coli* ∆*recA* ∆*recN* cells, co-expression of paRecA and paRecN fully restored MMC resistance, whereas co-expression of *E. coli* RecA (ecRecA) with paRecN conferred partial resistance to mitomycin C (MMC), demonstrating species-specific compatibility. Expression analysis revealed that paRecN was poorly expressed in *E. coli*, but codon optimization significantly enhanced its abundance and repair activity. We further identified gain-of-function paRecN mutants (I73T and R453H) that restored repair without increased expression. These mutants displayed species-specific adaptation, which improved compatibility with ecRecA but reduced functionality with paRecA. Fluorescence microscopy revealed that MMC-induced nucleoid localization was increased in paRecN^I73T^ and paRecN^R453H^ compared with paRecN. Collectively, these findings demonstrate that coevolution optimizes the RecA–RecN interface to ensure efficient DSB repair.

## Author summary

DNA double-strand breaks (DSBs) pose a serious threat to genomic integrity, and their accurate repair is essential for cell survival. In bacteria, DSB repair largely depends on homologous recombination mediated by the RecA recombinase and supported by the SMC-like protein RecN. Although RecA and RecN are known to cooperate, how their interaction contributes to efficient repair has remained unclear. Here, we investigated the functional relationship between RecA and RecN by expressing proteins from *Pseudomonas aeruginosa* in *Escherichia coli*. We found that *P. aeruginosa* RecN (paRecN) exhibits limited activity in *E. coli* but regains full functionality when co-expressed with its cognate RecA, revealing strong species-specific compatibility. Furthermore, we identified gain-of-function paRecN mutants that restore compatibility with *E. coli* RecA while losing compatibility with their native partner. These findings demonstrate that the RecA–RecN interaction interface is shaped by species-specific coevolution. Our study provides mechanistic and evolutionary insights into how protein–protein interactions involved in genome maintenance are optimized within each species.

## Introduction

DNA double-strand breaks (DSBs) arising from exogenous agents such as ionizing radiation or chemical mutagens or from endogenous processes such as replication fork blockage are among the most cytotoxic forms of DNA damage [1]. When left unrepaired or repaired inappropriately, DSBs can lead to deleterious genetic alterations and cell death. Homologous recombination (HR) is an evolutionarily conserved mechanism essential for accurate DSB repair that uses an intact double-stranded DNA molecule as a template [2–4].

In *Escherichia coli*, DSB ends are processed by the RecBCD complex, whose helicase and nuclease activities resect the broken DNA to produce 3′ single-stranded (ss)DNA overhangs [5–7]. Subsequently, RecA is loaded onto the ssDNA to form a nucleoprotein filament that mediates homology searching, homologous pairing, strand invasion, and strand exchange, ultimately generating a recombination intermediate between the broken DNA and an intact homologous template [8–14]. In addition, RecA-bound ssDNA acts as an SOS response signal by triggering the autocleavage of the transcriptional repressor LexA, activating the transcription of SOS genes involved in DNA repair, recombination, and cell division arrest [15]. Given the central role of RecA in DSB repair, the series of reactions it catalyzes must be closely related to the mechanism that controls chromosome integrity; however, how chromosome dynamics are coordinated through RecA-mediated DSB repair remains incompletely understood.

Structural maintenance of chromosomes (SMC) proteins are conserved from bacteria to humans and play essential roles in chromosome dynamics, including sister chromatid cohesion, chromosome condensation, transcription, and DNA repair [16–18]. The *E. coli recN* gene encodes an SMC-like protein that is induced as part of the SOS regulon [19]. *recN* mutants exhibit high sensitivity to ionizing radiation, I-*Sce*I-induced DSBs, and mitomycin C (MMC), but not to UV irradiation [20–22]. Moreover, ∆*recN* cells display abnormal nucleoid morphology characterized by fragmentation and/or decondensation in the presence of MMC [23], indicating a specific role for RecN in DSB repair. Consistent with this, we recently demonstrated that MMC-treated *recN*-deficient cells undergo a loss of chromosomal integrity and reduced survival, both of which are restored to wild-type levels upon subsequent induction of RecN expression [24]. Thus, the aberrant nucleoid structures observed in MMC-treated ∆*recN* cells do not represent irreversible chromosome disruption but rather a failure to complete RecA-mediated HR process. Fluorescence microscopy has shown that RecN is recruited to MMC-induced DSB sites in a RecA-dependent manner and plays a role in nucleoid compaction and sister chromatid interactions following DNA damage [23,25,26]. Recent live-cell imaging studies have demonstrated that RecA forms long elongated filaments on ssDNA (RecA bundles) that mediate pairing between DSBs and homologous donor strands located at distant sites, and that RecN modulates RecA filament dynamics to facilitate the search for homologous sequences [11,13,27]. *In vitro* studies have shown that RecN interacts with RecA to stimulate D-loop formation and strand-exchange activity [28,29]. As a cohesin-like protein, RecN can promote intermolecular DNA interactions and topologically entrap two DNA molecules in an ATP-dependent manner [29,30]. Together, these findings support that the SMC-like RecN plays structural and functional roles in HR-dependent DSB repair by coordinating RecA activity with high-order chromosome dynamics. Despite these advances, how RecN-mediated regulation of nucleoid dynamics contributes to the promotion of RecA-dependent HR, including the coordination of homology searching and strand exchange, remains poorly understood. Identifying the RecA–RecN interaction interface is therefore essential for elucidating the precise role of RecN in DSB repair.

In this study, we investigated the interaction between RecA and RecN in DSB repair using a cross-species functional complementation assay, expressing *Pseudomonas aeruginosa* RecA (paRecA) and RecN (paRecN) in *E. coli* ∆*recN* strain. paRecN displayed reduced DSB repair activity compared with *E. coli* RecN (ecRecN), but its activity was restored when co-expressed with paRecA. Furthermore, we isolated several paRecN mutants with enhanced DNA repair activity in *E. coli*, all of which carried mutations in either the N-terminal or the C-terminal domain. Interestingly, paRecN^I73T^ and paRecN^R453H^ showed repair activity comparable to that of ecRecN but lost their enhanced repair function when co-expressed with paRecA. These findings suggest that RecA–RecN interfaces are optimized through coevolution and highlight adaptive mechanisms restoring HR activity across species.

## Results

### Functional complementation of the ecRecN defect by RecN derived from other bacteria

**Fig 1:**
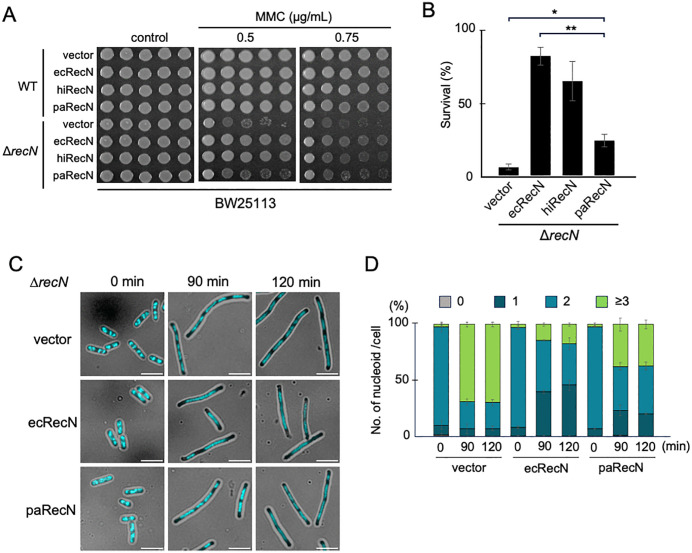
Functional complementation of paRecN in *E. coli.* **(A)** MMC sensitivity of WT and ∆*recN* strains expressing ecRecN, hiRecN, or paRecN. Ten-fold serial dilutions of cell cultures were spotted onto LB plates containing the indicated concentrations of MMC. Three independent experiments were performed which showed similar trends. **(B)** Exponentially growing Δ*recN* cells carrying the indicated plasmid were exposed to MMC (1.0 µg/mL) for 60 min. Aliquots were collected and plated on LB_Cm plates at appropriate dilutions. The survival rates were calculated as the number of viable cells relative to the control (0 min) samples. The data points represent the average of three independent experiments. Data shown are mean ± SEM. P values were determined using a two-tailed t -test. *P < 0.05, **P < 0.01. **(C)** Nucleoid morphology in ∆*recN* cells expressing ecRecN or paRecN following MMC treatment. Exponentially growing cells were treated with MMC (1.0 µg/mL) for 90–120 min, collected at the indicated time points, fixed, stained with DAPI, and observed by fluorescence microscopy. Nucleoids appear light blue. Scale bar = 5 μm. **(D)** Quantitative analysis of nucleoid morphology in **C**. The bar graph represents the percentages of cells with no nucleoid, one nucleoid, two nucleoids, and ≥ 3 nucleoids per cell. At least 100 cells were analyzed for each time point. Data are shown as mean ± SD from three independent experiments.

RecN is highly conserved among bacteria. A previous study showed that RecN (hiRecN) from *Haemophilus influenzae* —like *E. coli*, a member of γ-proteobacteria— restored MMC sensitivity in *E. coli* ∆*recN* to wild-type levels [31]. In the present study, we extended this comparative approach to *Pseudomonas aeruginosa* RecN (paRecN), another γ-proteobacterial RecN homolog but with lower sequence identity with ecRecN than hiRecN. To enable the expression of RecN from different species under comparable transcriptional control, we constructed plasmids in which either *H. influenzae recN* or *P. aeruginosa recN* was inserted downstream of the native *E. coli* SOS-inducible *recN* promoter. Cells of the BW25113 ∆*recN* strain were transformed with the plasmids and subjected to spot assays for MMC sensitivity. hiRecN and ecRecN exhibited similar MMC sensitivity, whereas paRecN conferred only partial MMC resistance compared to ecRecN (Fig 1A and 1B). Similar results were obtained in the AB1157 genetic background (S1A Fig). These results indicate that while paRecN retains substantial functionality in *E. coli*, it fails to achieve full DSB repair proficiency.

### Nucleoid morphology in paRecN-expressing ∆*recN* cells

One phenotypic hallmark of *E. coli* ∆*recN* is abnormal nucleoid morphology under DSB-induced genotoxic stress: RecN-deficient cells exposed to MMC exhibit nucleoid fragmentation and a filamentous morphology, reflecting incomplete repair [23,24,32]. To investigate whether paRecN can maintain nucleoid integrity under DNA-damaging conditions, we observed 4′,6-diamidino-2-phenylindole (DAPI)-stained nucleoids in MMC-treated *E. coli* ∆*recN* cells expressing ecRecN, paRecN, or an empty vector. Following MMC treatment, ∆*recN* cells expressing ecRecN exhibited elongated, evenly spaced nucleoids aligned along the cell axis; however, the number of nucleoids per cell remained similar to that in untreated controls, typically with one or two per cell (Fig 1C and 1D). In contrast, ∆*recN* cells carrying the empty vector exhibited extensive filamentation and nucleoid fragmentation, with 70% of cells having three or more nucleoids per cell after 120 min of MMC treatment (Fig 1C and 1D). In paRecN-expressing cells, this proportion was reduced to 38% (Fig 1C and 1D), indicating a partial loss of functionality in maintaining nucleoid integrity.

### DNA repair activity in paRecA and paRecN co-expressing cells

**Fig 2:**
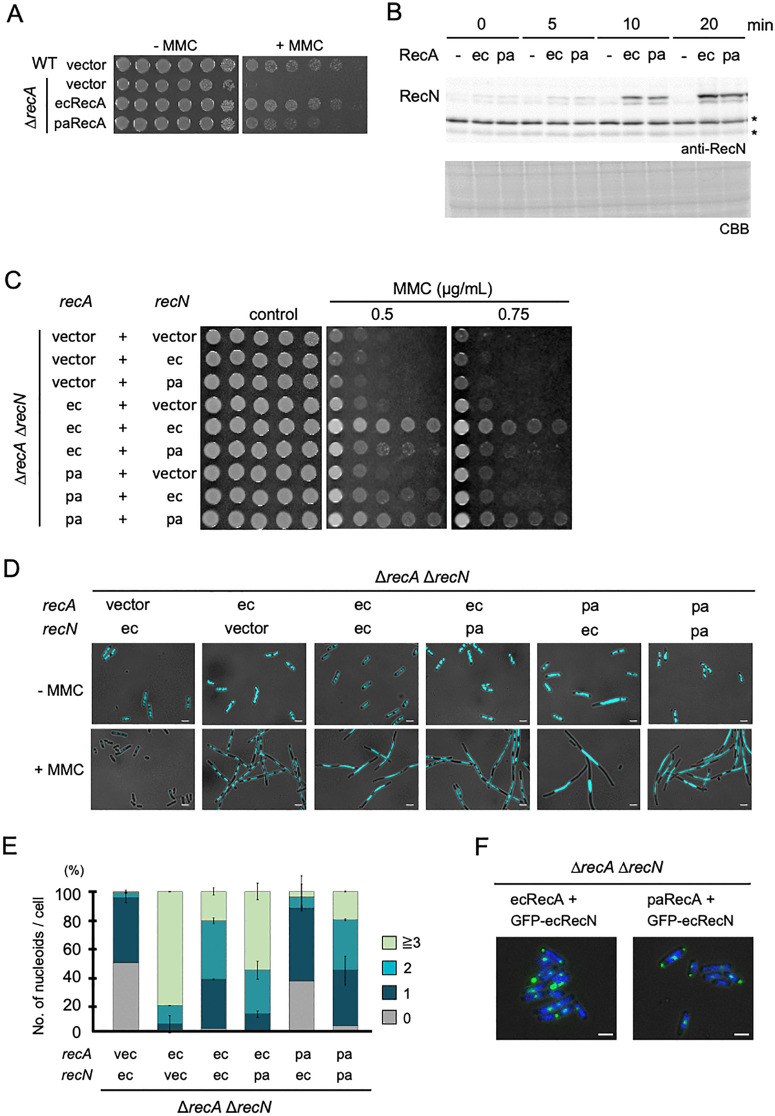
Functional complementation in cells co-expressing paRecA and paRecN. **(A)** MMC sensitivity of ∆*recA* strains expressing ecRecA or paRecA. Ten-fold serial dilutions were spotted onto LB plates in the presence or absence of MMC (0.5 μg/mL) and incubated at 37 °C. Three independent experiments were performed which showed similar trends. (**B**) paRecA is proficient in SOS response induction. Cells were treated with MMC (1.0 µg/mL) for 20 min, collected at the indicated time points. Protein extracts were analyzed by western blotting using an anti-RecN antibody. Asterisk indicates a non-specific band (upper panel). Coomassie Brilliant Blue (CBB) stain is used as a loading control (lower panel). Two independent experiments were performed which showed similar trends. **(C)** MMC sensitivity of ∆*recA* ∆*recN* cells co-expressing RecA and RecN from the indicated species. Spot assays were performed as in **A**. ec, *E. coli*; pa, *P. aeruginosa*. Four independent experiments were performed which showed similar trends. **(D)** Cell morphology and nucleoid organization in MMC-treated ∆*recA* ∆*recN* cells co-expressing RecA and RecN. Images of DAPI-stained of cells treated or not with MMC (1.0 μg/mL) for 120 min. Nucleoids appear light blue. Scale bar = 2.5 μm. **(E)** Quantitative analysis of nucleoid morphology in **D**. The bar graph represents the percentages of cells with no nucleoid, one nucleoid, two nucleoids, and ≥ 3 nucleoids per cell. At least 100 cells were analyzed per condition. Data are shown as mean ± SD from three independent experiments. **(F)** ∆*recA* ∆*recN* cells expressing GFP-ecRecN and either ecRecA or paRecA were treated with MMC (0.75 μg/mL) for 30 min. Panels show GFP/DAPI/bright-field-merged cell images. Scale bar = 2.5 μm.

Given the established biochemical interaction between RecA and RecN in several bacteria [28,29], we speculated that the reduced activity of paRecN in *E. coli* could result from an inefficient interaction with ecRecA. To explore this possibility, we examined whether co-expression of paRecA and paRecN would restore survival. We first evaluated the ability of paRecA to replace ecRecA function in *E. coli*. To this end, we generated a paRecA expression plasmid by replacing the *E. coli recA* coding sequence with that of *P. aeruginosa*. Expression of ecRecA in ∆*recA* cells restored survival under MMC exposure to wild-type levels (Fig 2A). In contrast, ∆*recA* cells expressing paRecA exhibited intermediate resistance (Fig 2A). Because RecA functions in both HR repair and SOS induction, we next examined whether the partial defect of paRecA in MMC resistance reflected an inability to induce the SOS response. We monitored SOS-regulated gene induction by monitoring RecN protein levels after MMC treatment. Strikingly, ∆*recA* cells expressing either ecRecA or paRecA showed comparable induction kinetics and RecN expression levels following MMC exposure (Fig 2B), indicating that paRecA is proficient in inducing the SOS response. These suggest that paRecA partially loses its HR repair activity in *E. coli*.

We next co-expressed RecA and RecN in a ∆*recA* ∆*recN* double mutant strain. Two compatible plasmids were employed: (i) pSTV28-based vectors for the expression of either ecRecA or paRecA under the native *E. coli recA* promoter, and (ii) pSCH19-based vectors for ecRecN or paRecN expression driven by the *E. coli* SOS-inducible *recN* promoter. Remarkably, in cells co-expressing paRecA and paRecN, MMC survival was significantly restored when compared with that of cells with heterologous combinations of ecRecA and paRecN or paRecA and ecRecN (Fig 2C). This implies that paRecN interacts more effectively with paRecA than with ecRecA. Identical trends were observed in the AB1157 genetic background (S1B Fig), underscoring the generality of the species-specific interaction effect.

### Nucleoid structure in paRecA and paRecN co-expressing cells

Next, we investigated nucleoid organization in *E. coli* ∆*recA* ∆*recN* cells co-expressing RecA and RecN. Cells were treated with MMC for 120 min, stained with DAPI and examined using fluorescence microscopy. Cells co-expressing ecRecA and ecRecN became highly filamentous, with elongated and evenly spaced nucleoids (Fig 2D), closely resembling the morphology of wild-type cells after MMC treatment. In contrast, ecRecA-paRecN co-expressing cells displayed an increased proportion of fragmented nucleoids, resembling the phenotype of ∆*recN* cells (Fig 2D and 2E). Cells co-expressing paRecA and ecRecN exhibited features similar to those of ∆*recA* cells, including an increased proportion of anucleate cells and cells containing only a single nucleoid (Fig 2E). However, in contrast to ∆*recA* cells, which did not exhibit cell elongation, paRecA-ecRecN co-expressing cells became filamentous, and the nucleoids were centrally condensed within the elongated cells (Fig 2D), suggesting the incomplete resolution of recombination intermediates. Similar to ecRecA, paRecA was able to induce nucleoid-associated ecRecN foci in the presence of MMC, (Fig 2F), indicating that it can recruit ecRecN to DSB sites. These results suggest that although paRecA supports a normal SOS response and can promote early HR steps, defects in HR repair compromise the resolution of recombination intermediates (see discussion). Notably, paRecA-paRecN co-expressing cells exhibited nucleoid morphologies largely indistinguishable from those of ecRecA-ecRecN cells (Fig 2D and 2E), supporting that functional interactions are preserved via species-specific associations during the repair process.

### Screening and isolation of gain-of-function paRecN mutants

**Fig 3:**
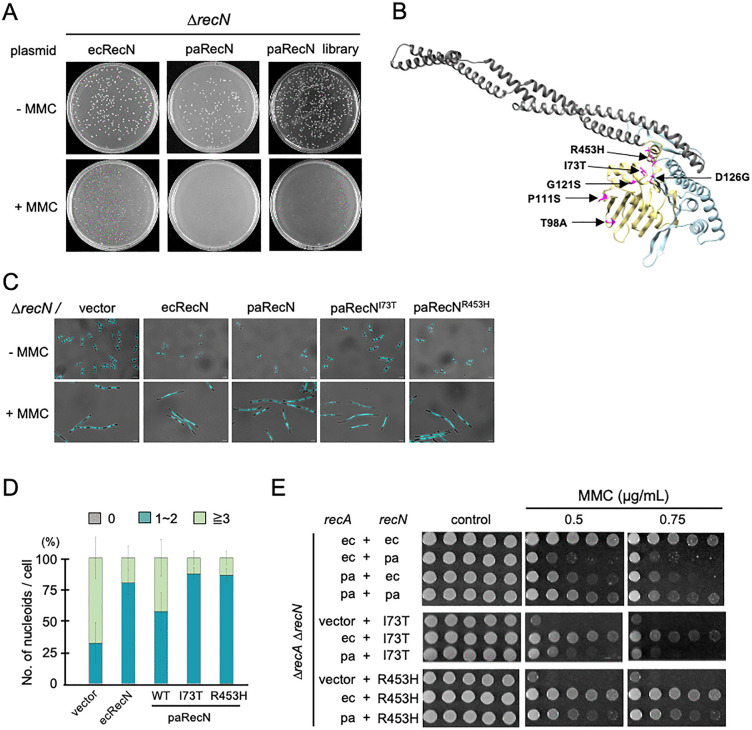
Screening of gain-of-function paRecN mutants. **(A)** Representative screening plates. ∆*recN* cells transformed with a paRecN mutant library were plated on LB agar in the presence or absence of MMC (0.5 µg/mL). The plates were incubated overnight at 37 °C. **(B)** Structural mapping of mutation sites on the paRecN model. The structure of paRecN shown in ribbon representation was predicted using AlphaFold2. Gain-of-function mutations identified in this study are highlighted in magenta. N-terminal and C-terminal domains are shown in gold and cyan, respectively. **(C)** Nucleoid morphology in MMC-treated ∆*recN* cells expressing paRecN mutants. Exponentially growing cells were treated or not with MMC (1.0 µg/mL), fixed, stained with DAPI, and analyzed using fluorescence microscopy. Panels show DAPI images of cells treated or not with MMC for 120 min. Scale bar = 2.5 μm. **(D)** Quantitative analysis of nucleoid morphology in **C**. The bar graph represents the percentages of cells with no nucleoid, one or two nucleoids, and ≥ 3 nucleoids per cell. At least 100 cells were analyzed for each condition. Data are shown as mean ± SD from three independent experiments. **(E)** MMC sensitivity of ∆*recA* ∆*recN* cells co-expressing RecA and RecN from the indicated species. Ten-fold serial dilutions of cell cultures were spotted onto LB plates containing the indicated concentrations of MMC. Four independent experiments were performed which showed similar trends.

Given the importance of species-specific RecA–RecN interaction for repair efficiency, we reasoned that targeted changes in either paRecN or paRecA could enhance their ability to function in *E. coli*. We chose to focus on RecN because it is primarily involved in DNA damage repair, whereas RecA is a multifunctional protein essential for homologous recombination, the SOS response, and cell viability. Therefore, we aimed to identify gain-of-function mutations in paRecN that restore DNA repair activity in *E. coli.* A paRecN mutant library created via PCR random mutagenesis was transformed into ∆*recN* cells, which were then plated onto Luria–Bertani (LB) agar containing MMC. Growing colonies were isolated as candidate gain-of-function mutants (Fig 3A). To confirm that improved MMC resistance was attributable to changes in paRecN, plasmids were recovered from each resistant clone and reintroduced into fresh ∆*recN* cells for retesting. We thus obtained 24 independent mutants showing reproducible MMC resistance (S2A Fig). Sequencing of the 24 *recN*-coding regions revealed 10 single amino-acid substitutions (comprising six unique mutations) and 11 double substitutions (comprising nine unique combinations) (S2A Fig). For three mutants, no mutations were detected in the coding sequence, suggesting possible changes in regulatory regions or extragenic suppressors. Mapping of the six single substitutions onto the primary RecN sequence revealed that five were located in the N-terminal domain, and one in the C-terminal domain. None of the altered residues were conserved between paRecN and ecRecN (S2B Fig), suggesting their potential role in species-specific functional adaptation.

### Functional complementation of paRecN gain-of-function mutants

When the six single substitutions identified were mapped onto the AlphaFold2-predicted structure of paRecN, the mutations were distributed across distinct structural regions (Fig 3B). Some substitutions were located on the outer surfaces of the globular domains and were spatially separated from one another, whereas others mapped near the interface between the N- and C-terminal domains. These observations suggest that multiple, potentially distinct mechanisms may underlie the functional restoration of paRecN in *E. coli*. Notably, I73T and R453H were found to be located in close spatial proximity despite their distant location in the primary sequence (Fig 3B), raising the possibility that both residues contribute to a shared functional surface. Based on this observation, we focused subsequent analysis on these two mutants and performed detailed functional characterization to evaluate the extent and specificity of their activity in *E. coli*. To determine whether this enhanced functionality correlated with improved nucleoid integrity under DNA-damaging conditions, we observed nucleoid structures in ∆*recN* cells expressing paRecN^I73T^ or paRecN^R453H^. Following 120 min of MMC treatment, the cells became filamentous while maintaining almost the same number of nucleoids per cell, a phenotype similar to that of wild-type *E. coli* (Fig 3C and 3D). These results confirmed that the I73T and R453H substitutions restored the ability of paRecN to maintain nucleoid integrity during MMC exposure, re-establishing functional activity in *E. coli* to the wild-type level.

Based on evidence regarding the species-specific compatibility between RecA and RecN, we next tested whether the improved function of paRecN^I73T^ and paRecN^R453H^ depended on the presence of ecRecA. Each mutant protein was co-expressed with ecRecA or paRecA in ∆*recA* ∆*recN* cells, which were then tested for MMC sensitivity. As expected, both mutants fully complemented the MMC sensitivity of the ∆*recA* ∆*recN* strain in the presence of ecRecA (Fig 3E). Remarkably, when coexpressed with paRecA, the ability of these paRecN mutants to confer MMC resistance was substantially reduced compared with that of wild-type paRecN (Fig 3E). This finding suggests that these mutations represent species-specific adaptive mutations that selectively enhance functional interaction with ecRecA at the expense of compatibility with paRecA.

### Effect of the paRecN expression level on functional complementation

**Fig 4:**
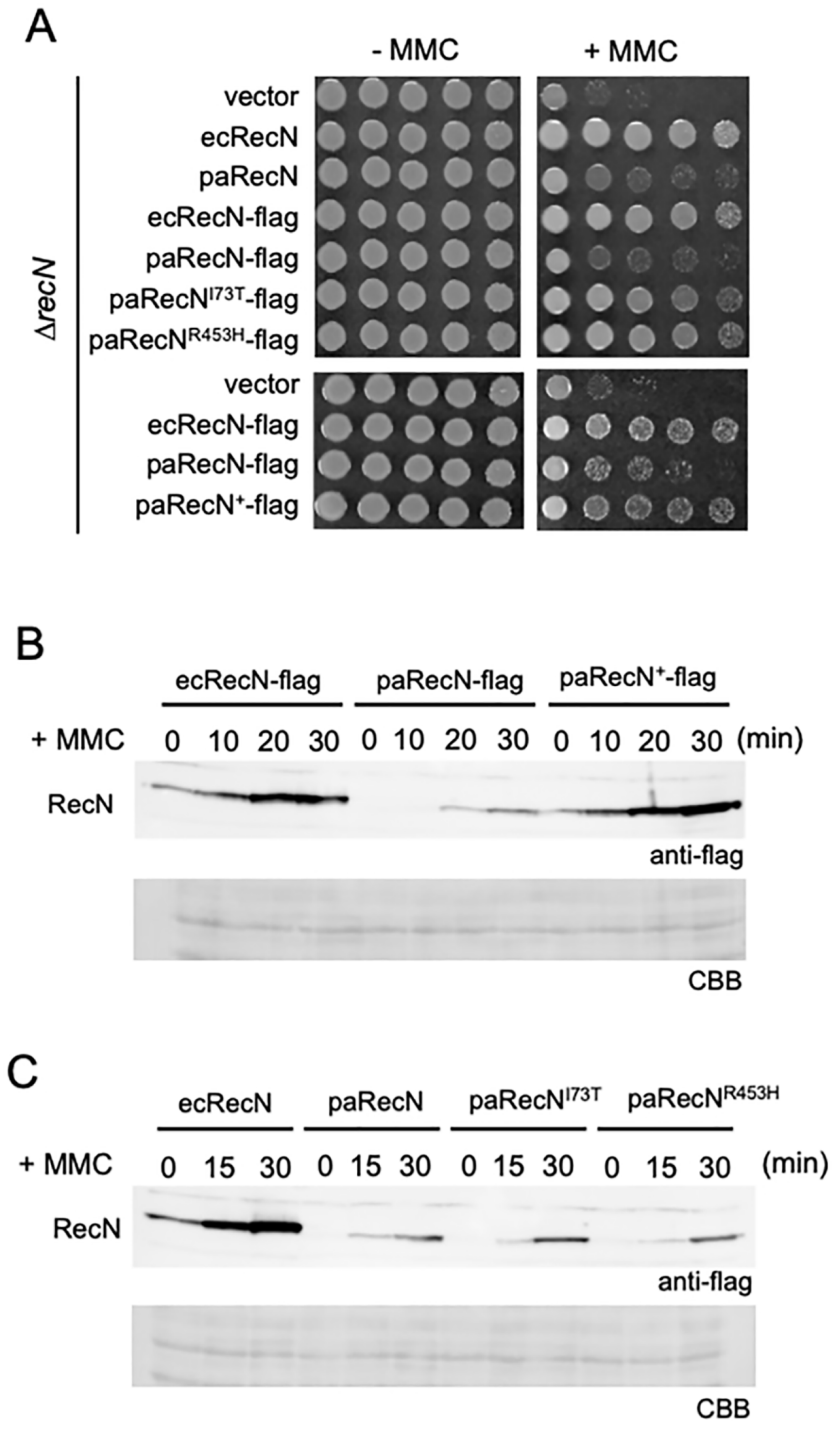
Expression levels of paRecN and its mutants. **(A)** MMC sensitivity of ∆*recN* cells expressing C-terminally Flag-tagged ecRecN, paRecN, or paRecN mutants. Ten-fold serial dilutions were spotted onto LB plates in the presence or absence of MMC (0.75 μg/mL). Three independent experiments were performed which showed similar trends. **(B)** ∆*recN* cells expressing ecRecN, paRecN, or codon-optimized paRecN (paRecN^+^) were treated with MMC (1.0 μg/mL) for the indicated times. Protein extracts were prepared and analyzed using western blot with an anti-flag antibody (upper panel). Coomassie stain was used as a loading control (lower panel). Three independent experiments were performed which showed similar trends. **(C)** MMC-dependent induction of paRecN mutant proteins. Detection was performed as in **B**.

To examine the expression level of paRecN, we constructed C-terminally Flag-tagged versions of ecRecN, paRecN, paRecN^I73T^, and paRecN^R453H^, and confirmed that the addition of the flag tag did not alter MMC resistance phenotypes in *E. coli* (Fig 4A). Western blot analysis of MMC-treated cells revealed that wild-type paRecN was expressed at a substantially lower level than ecRecN, despite being driven by the same promoter (Fig 4B), suggesting that sequence-intrinsic factors, such as rare codons, might impair translation efficiency in *E. coli*. To evaluate this hypothesis, we synthesized a codon-optimized version of paRecN (paRecN^+^), replacing infrequently used codons with synonymous codons preferred in *E. coli* (S3 Fig). The codon-optimized paRecN^+^ was expressed at a level comparable to that of ecRecN and fully complemented MMC resistance (Fig 4A and 4B), confirming that reduced expression of paRecN leads to its decreased functionality in *E. coli* cells. Importantly, both paRecN^I73T^ and paRecN^R453H^ were expressed at low levels similar to that of wild-type paRecN (Fig 4C), indicating that their functional restoration is not attributable to increased protein expression, but rather to qualitative improvements in their function, likely in terms of protein–protein interactions and/or activity, conferred by the amino-acid substitutions.

### Function of GFP-tagged paRecN

**Fig 5:**
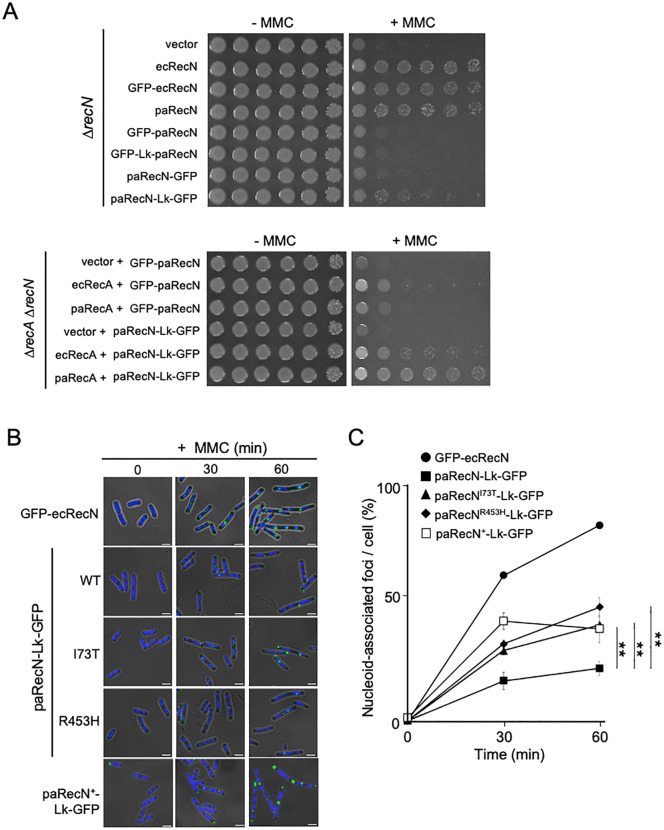
Cellular localization and function of paRecN and its mutants. **(A)** Upper panel: MMC sensitivity of ∆*recN* cells expressing GFP fusions at the N- or C-terminus of RecN. Lower panel: MMC sensitivity of ∆*recA* ∆*recN* cells co-expressing paRecA and paRecN fused to GFP at the N- or C-terminus. Ten-fold serial dilutions were spotted onto LB plates in the presence or absence of MMC (0.5 μg/mL). Three independent experiments were performed which showed similar trends. **(B)** ∆*recN* cells expressing GFP-ecRecN, paRecN-Lk-GFP, paRecN mutant-Lk-GFP, or paRecN^+^-Lk-GFP were treated with MMC (0.75 μg/mL), fixed, stained with DAPI, and analyzed using fluorescence microscopy. Panels show merged GFP/DAPI/bright-field images. Nucleoids appear dark blue. Scale bar = 2.5 μm. **(C)** Quantitative analysis of nucleoid-associated GFP-RecN foci in **B**. At least 100 cells were analyzed for each time point. Data are shown as mean ± SD from three independent experiments. Significance difference was assessed by Student’s *t*-test. **P < 0.001.

Previous studies showed that N-terminally GFP-tagged ecRecN (GFP-ecRecN) fusion protein retains full DNA repair activity and forms discrete foci on nucleoids after MMC treatment (Fig 5A and 5B) [23,32,33]. To assess whether a similar strategy would allow visualization of paRecN, we constructed an N-terminal GFP-paRecN fusion driven by the SOS-inducible *recN* promoter (S4A Fig). However, GFP-paRecN showed the same MMC sensitivity as ∆*recN* cells, indicating a loss of function (Fig 5A). Similar N-terminal tagging effects have been reported for *Bacillus subtilis* RecN [23,25,26], underscoring species-specific structural features of RecN. Therefore, we created alternative fusion configurations to minimize steric hindrance, including GFP-Lk-RecN, in which GFP was fused to the N-terminus via a flexible linker (Lk), and RecN-GFP or RecN-Lk-GFP, in which GFP was fused to the C-terminus either directly or via the flexible linker (S4A Fig). When expressed in ∆*recN* cells, both GFP-Lk-paRecN and paRecN-GFP failed to complement MMC sensitivity (Fig 5A). In contrast, paRecN-Lk-GFP conferred partial MMC resistance, albeit with reduced survival compared to paRecN (Fig 5A). Furthermore, co-expression of paRecA with paRecN-Lk-GFP, but not with GFP-paRecN, in ∆*recA* ∆*recN* cells restored MMC resistance (Fig 5A). These results indicate that C-terminal Lk fusion is the least disruptive configuration for paRecN functionality in *E. coli*.

### Intracellular localization of RecN-Lk-GFP

To investigate whether functional differences correlated with the subcellular localization of RecN, we analyzed the formation of nucleoid-associated foci following MMC treatment in ∆*recN* cells expressing either GFP-ecRecN, paRecN-Lk-GFP, or codon-optimized paRecN (paRecN^+^)-Lk-GFP. As reported previously, distinct GFP-ecRecN foci formed on the nucleoid 30 min after MMC treatment, and the proportion of cells exhibiting nucleoid-associated foci increased to approximately 80% by 60 min (Fig 5B and 5C). In contrast, only ~20% of cells expressing paRecN-Lk-GFP exhibited nucleoid-associated foci at 30 min, and this proportion did not increase at 60 min (Fig 5B and 5C). The weak fluorescence intensity of paRecN-Lk-GFP likely reflects its reduced expression level, as confirmed by western blotting with anti-GFP antibodies (S4B Fig). Consistent with this, paRecN^+^-Lk-GFP exhibited stronger fluorescence, reflecting increased expression. In addition, paRecN^+^ showed a substantially higher proportion of nucleoid-associated foci than wild-type paRecN (Fig 5B and 5C). These results suggest that increased protein abundance can partially restore nucleoid localization.

Although paRecN-Lk-GFP cannot be directly compared to GFP-ecRecN due to differences in fusion constructs and expression patterns, these observations suggest that paRecN displays reduced nucleoid localization in response to DNA damage. We therefore evaluated the formation of nucleoid-associated foci by paRecN^I73T^ and paRecN^R453H^. As expected, both paRecN^I73T^-Lk-GFP or paRecN^R453H^-Lk-GFP were expressed at lower levels, similar to that of wild-type paRecN-Lk-GFP, but conferred higher MMC resistance than paRecN-Lk-GFP (S4B and S4C Fig). Microscopy analysis revealed that, during MMC treatment, the proportion of cells containing nucleoid-associated foci was increased in both mutants relative to wild-type paRecN-Lk-GFP and reached levels comparable to those observed for the paRecN^+^ (Fig 5B and 5C). Taken together, these results suggest that both increased protein abundance (paRecN^+^) and amino acid substitutions (paRecN^I73T^ and paRecN^R453H^) can enhance nucleoid localization, although potentially through distinct mechanisms. The gain-of-function mutants, in particular, may promote more efficient recruitment to, and/or altered interactions with, the nucleoid in response to DNA damage, which may contribute to their enhanced repair activity in *E. coli*.

### RecA-RecN interaction

Previous studies have shown that ecRecN physically interacts with ecRecA [28,29]. To examine the interaction between ecRecA and paRecN or its mutants, we purified paRecN and its variants. During purification, we found that the N-terminally His-tagged construct (His-paRecN) failed to bind to a Ni² ⁺ affinity column. This observation suggests that N-terminal tagging interferes with paRecN function, consistent with our previous results using GFP fusion constructs. Therefore, paRecN was purified as a C-terminally His-tagged protein (S5 Fig).

**Fig 6:**
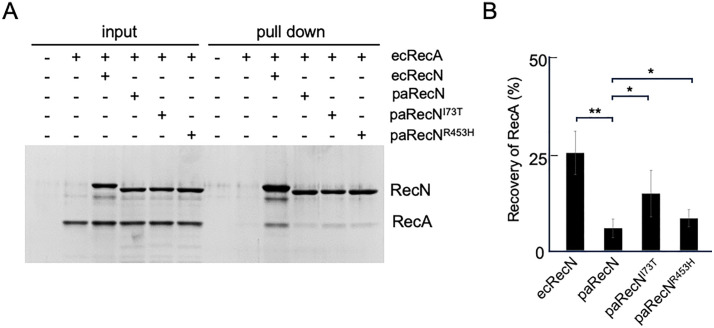
The physical interaction of ecRecN/paRecN with ecRecA. **(A)** The reaction mixtures were incubated in the presence of the indicated proteins (2 μM each protein). The proteins bound to Co^2+^-conjugated beads were collected, washed, eluted with SDS–sample buffer, and then analyzed by SDS–PAGE and CBB staining. **(B)** The RecA/RecN ratio in the pull-down samples was quantified using ImageJ software. The data shown in (**A**) represent the mean ± SD from three independent experiments. P values were determined using a Student’s *t*-test (*P < 0.05, **P < 0.01).

Using purified ecRecA, ecRecN, and paRecN proteins, we performed pull-down assays to assess their binding interactions. Following incubation of ecRecA with RecN, RecA–RecN complexes were recovered using Co² ⁺ -conjugated magnetic beads and analyzed by SDS–PAGE. Under these conditions, His-ecRecN, paRecN-His, paRecN^I73T^-His and paRecN^R453H^-His were recovered at comparable levels on the beads (Fig 6A). In contrast, the amount of ecRecA co-recovered with paRecN was markedly reduced compared with that recovered with ecRecN (Fig 6A and 6B). Notably, the paRecN^I73T^ and paRecN^R453H^ mutants showed a modest increase in ecRecA recovery relative to wild-type paRecN (Fig 6A and 6B). These results indicate that paRecN exhibits a reduced physical interaction with ecRecA compared with ecRecN, and that the paRecN mutant partially restores this interaction.

## Discussion

In this study, we investigated the functional interplay between RecA and RecN during DSB repair in *E. coli* using functional complementation assays with *P. aeruginosa* RecA and RecN*.* We found that neither paRecA nor paRecN alone could fully rescue the MMC sensitivity of *E. coli*, whereas co-expression of both proteins did restore survival. These results suggest that the reduced DNA repair activity of the heterologous proteins in *E. coli* arises from a defect in the functional interaction between the two proteins originating from different species.

*E. coli* ∆*recA* cells expressing paRecA showed only partial resistance to MMC, yet retained the ability to activate the SOS response, as indicated by SOS gene induction and MMC-dependent filamentation. This suggests that paRecA is impaired in HR repair rather than in SOS response. In this regard, cells expressing paRecA exhibited abnormal nucleoid morphologies in response to DNA damage, including highly condensed nucleoids and an increased frequency of anucleate cells. This phenotype resembles that of ∆*ruvC*, but not ∆*recA*, which exhibit a filamentous phenotype with centrally located chromosome aggregates and subsequently produced anucleate cells [34]. Given that RuvC is essential for resolving Holliday junction intermediates formed by RecA [35,36] and that paRecA can promote the formation of nucleoid-associated ecRecN foci, these results suggest that while paRecA can recruit ecRecN to DSB sites and promote early steps of HR, such as sister chromatid interaction and D-loop formation, it may not fully support the functional interaction between paRecA and ecRecN that is required for the later stages of recombination, leading to the accumulation of unresolved recombination intermediates.

**Fig 7:**
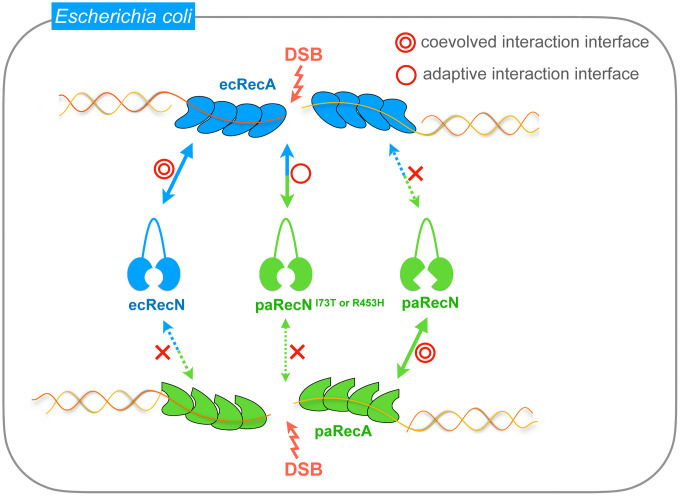
The species-specific coevolution of RecA–RecN interfaces in DSB repair. In *Escherichia coli*, ecRecN is recruited to DSB sites in a RecA-dependent manner through a coevolved interaction interface with ecRecA. In contrast, paRecN preferentially interacts with paRecA via a species-specific coevolved interface and therefore shows limited functionality in the *E. coli* cellular context. Gain-of-function paRecN mutants (I73T or R453H) acquire an adaptive interaction interface that restores compatibility with ecRecA. However, this adaptive interaction is accompanied by a loss of compatibility with paRecA, highlighting a trade-off imposed by species-specific coevolution of protein–protein interaction interfaces.

We found that paRecN was expressed at lower levels than ecRecN. Codon optimization for *E. coli* significantly improved paRecN expression and fully restored MMC resistance in ∆*recN* cells, indicating that increased protein abundance can compensate for inefficient interaction between ecRecA and paRecN. Beyond this expression effect, however, the gain-of-function mutants paRecN^I73T^ and paRecN^R453H^ conferred MMC resistance without any increase in protein levels. Notably, these mutants gained compatibility with ecRecA while losing that with paRecA, demonstrating that the substitutions re-established a species-specific interaction optimized for *E. coli* (Fig 7). We also found that MMC-induced nucleoid localization was increased in paRecN^I73T^ and paRecN^R453H^ compared with paRecN, suggesting that their enhanced repair activity may be attributable to improved recruitment to DNA damage sites. Taken together, these findings demonstrate that species-specific RecA–RecN compatibility likely contributes to efficient DSB repair in *E. coli*, and that enhanced recruitment of the mutant paRecN proteins to damage sites plays a central role in their functional restoration (Fig 7). Furthermore, the interaction between ecRecA and paRecN was markedly reduced compared with that of the ecRecA–ecRecN pair, whereas paRecN^I73T^ and paRecN^R453H^ showed a modest but reproducible recovery in their interaction with ecRecA relative to paRecN. This partial restoration may reflect an improved compatibility at the ecRecA–paRecN interface. However, because the binding of these mutants to ecRecA remains substantially weaker than that of ecRecN, it remains unclear to what extent functional recovery can be attributed solely to this effect. Other factors, such as interactions with additional factors or recovery of functional (rather than strictly physical) interactions with ecRecA, may also contribute to the observed phenotypes. Notably, although the two substituted residues are distant in the primary sequence, they are located in close proximity at the interface between the N- and C-terminal domains implicated in ATP binding. Previous study has shown that DNA-bound RecA can stimulate RecN ATP hydrolysis in a species-specific manner [28]. This raises the possibility that, in addition to enhanced nucleoid recruitment, altered RecN ATPase activity in paRecN^I73T^ and paRecN^R453H^ may also contribute to their functional recovery, leaving this as an intriguing but unverified possibility that warrants further investigation.

Protein coevolution is a process in which pairs of proteins undergo coordinated changes, generally to preserve or optimize their functional association; therefore, protein–protein interaction interfaces are often under coevolutionary constraints [37]. In this study, we identified species-specific gain-of-function paRecN mutants that regained functional interaction with ecRecA while concomitantly losing compatibility with paRecA. Notably, none of the substitutions identified occurred at conserved residues. These findings indicate that the RecA–RecN interface required for functional interaction is not universally conserved across species but has likely been optimized through species-specific coevolution driven by the multifunctional nature of RecA and RecN. Indeed, the SMC-like RecN not only cooperates with RecA during HR but also contributes to high-order chromosome organization and dynamics. Given that the bacterial nucleoid comprising genomic DNA, RNA, and nucleoid-associated proteins is a highly dynamic and species-specific structure that adapts to environmental fluctuations [38–40], it is plausible that the RecN–RecA interface has coevolved in parallel with the species-specific evolution of nucleoid organization.

Our findings indicate that RecA–RecN interaction is essential for DSB repair while being subject to species-specific constraints. These results suggest that coevolution may optimize the RecA–RecN interface within each species to support efficient DSB repair. The gain-of-function paRecN mutants identified in this study were localized to the N- and C-terminal globular domains. Notably, some of these substitutions are spatially separated within the structure, suggesting that functional restoration may not be explained solely by improved RecA–RecN interface interactions. Instead, multiple mechanisms may contribute to the recovery of DNA repair activity in *E. coli*, including partial restoration of RecA interaction, functional modulation of RecN, or altered interactions with additional factors. These findings may reflect an adaptive mechanism that enables paRecN to function with a heterologous RecA. Future studies should focus on biochemical characterization of these mutants, as well as the additional mutants not examined here, to dissect the relative contributions of RecA interaction and ATP hydrolysis to HR promotion. A better understanding of these mechanisms may provide further insight into the structural and functional roles of RecN in HR-dependent DSB repair, which likely requires coordination between RecA and chromosome dynamics.

## Materials and methods

### Bacterial strains and plasmids

All strains used in this study were derivatives of BW25113, unless otherwise stated, and are listed in Table 1. *H. influenzae* and *P. aeruginosa recN* genes were PCR-amplified from genomic DNA from *H. influenzae* strain Rd and *P. aeruginosa* strain PAO1 (American Type Culture Collection), respectively. An *E. coli recN* fragment containing the native SOS promoter was cloned into pSTV28 and pSCH19, generating pST-RecN and pSC-RecN, respectively. The *recN* genes in pST-RecN and pSC-RecN were replaced with *P. aeruginosa* or *H. influenzae recN* to generate pST-paRecN, pST-hiRecN, and pSC-paRecN. An *E. coli recA* fragment containing the native promoter was cloned into pSTV28, generating pST-RecA. Plasmids expressing paRecA were constructed by replacing the *E. coli recA* open reading frame in pST-RecA and pRecA [23] with *P. aeruginosa recA*, yielding pST-PaRecA and pPaRecA. C-terminally flag-tagged RecN was constructed from pST-RecN/pST-paRecN by introducing a Flag epitope coding sequence into the 3′ end of *recN* in frame. RecN/paRecN proteins were tagged with an enhanced GFP cassette at either the N- or C-terminus in pST-RecN or pST-paRecN, generating pGFP-RecN/paRecN and pRecN/paRecN-GFP. For pGFP-Lk-RecN/paRecN, a linker sequence (GGATCCGCTGGCTCCGCTGCTGGTTCTGGCGAATTCCAT) was inserted between GFP and the recN N-terminus. For pRecN/paRecN-Lk-GFP, a linker sequence (GGCGGAGAATTCTTGGATTCAA-TAGAAAAGGTAAGCGAATTTGCCACC) was inserted between GFP and the RecN C-terminus. For the codon-optimized paRecN plasmid, we used a codon optimization tool provided by Gene Frontier that redesigned *P. aeruginosa recN* by substituting rare codons with synonymous codons preferred in *E. coli*. The optimized open reading frame gene of paRecN was synthesized and cloned downstream of the SOS promoter of *recN* in pSTV28, yielding pST-paRecN^+^. All *recN* mutants were generated by PCR-based mutagenesis and used to substitute wild-type *recN* on each plasmid. Recombinant plasmid structures were confirmed by DNA sequencing.

**Table 1:** *E. coli* strains used in this study.

Strain	Genotype	Source
BW25113	*rph-1*, Δ*lacZ4787*, Δ*(rhaBAD)568*, Δ*(araBAD)567*, *rrnB*, *HsdR514*	NBRP
JW2669-KC	BW25113, Δ*recA*::*Km*	NBRP
JW5416-KC	BW25113, Δ*recN*::*Km*	NBRP
SN001	BW25113, Δ*recA*::*Km*, Δ*recN15002*::*Tn5*	[24]
AB1157	*thr-1*, *leuB6*, *thi-1*, *tsx-33*, *sup-37*, *supE44*, *lacY1*, *galK2*, *ara-14*, *xyl-5*, *mtl-1*, *proA2*, *his-4*, *argE3*, *rpsL31*	[41]
AMI001	AB1157, Δ(srl-*recA)306*::*Tn10*	[34]
HRS2006	AB1157, Δ*recN15002*::*Tn5*	Lab stocks
AMI002	AB1157, Δ(srl-*recA)306*::*Tn10*, Δ*recN15002*::*Tn5*	This study

### Media and general methods

For *E. coli* genetic manipulation and DNA recombination, standard methods as described by Miller and Sambrook were used [42,43]. Ampicillin (50 μg/mL), tetracycline (10 μg/mL), chloramphenicol (100 μg/mL), and kanamycin (30 μg/mL) were added to Luria Bertani (LB) broth as required. MMC (0.5 mg/mL stock) was dissolved in 10 mM Tris-HCl (pH 8.5).

### Sensitivity to MMC

The cultures were grown in LB broth to an A_600_ of ~0.4. They were serially diluted and spotted onto LB containing ampicillin and/or chloramphenicol, where appropriate, and the indicated concentration of MMC. The plates were then incubated at 37 °C overnight.

### Isolation of paRecN mutants

PCR-mediated random mutagenesis was carried out on pSC-RecN using forward and reverse oligonucleotides; paRecNF (GCCGCCCCATATGCTGGTTCACCTGTCCGTTCAC) and paRecNR (GGAAAGGATCCGCGAGCGCGGCTTCGAGCTGGTCG) containing *Nde*I and *Bam*HI sites, respectively. A library of paRecN mutants was generated by replacing mutagenized *Nde*I-*Bam*HI fragments with wild-type *P. aeruginosa recN* in pSC-paRecN. The AB1157 ∆*recN* strain was used for screening because it has a wider dynamic range for detecting improvement than the BW25113 ∆*recN* strain. The resultant paRecN mutant library was transformed into a ∆*recN* strain, and transformants were plated on LB agar supplemented with 0.5 μg/mL MMC and ampicillin. MMC-resistant colonies were selected after overnight incubation.

### *E. coli* cell extract and western blotting

Cells were grown at 37 °C to an OD_600_ of 0.5 and treated with 0.5 or 1.0 μg/mL MMC at 37°C. Aliquots were taken at the indicated times and centrifuged. Cells were rapidly frozen in liquid nitrogen, resuspended in SDS loading buffer (82 mM Tris-HCl at pH 6.8, 5.6 M urea, 1.6 M thiourea, 0.24 M NaCl, 2% SDS, 0.1% bromophenol blue, 10% glycerol, and 2% 2-mercaptoethanol), and lysed by boiling. Samples were analyzed by 10% SDS-polyacrylamide gels, and proteins were transferred to polyvinylidene difluoride (PVDF) membranes. ecRecN, RecN-flag and RecN-GFP were detected with anti-ecRecN [33], anti-flag (Sigma), and anti-GFP (MBL) antibodies, respectively. The protein bands were visualized by enhanced chemiluminescence detection (Western blot ultrasensitive horseradish peroxidase [HRP] substrate; Takara). Coomassie brilliant blue stain was used as a loading control.

### SOS response

SOS response induction was monitored by measuring RecN expression, as described previously. Overnight cultures of ∆*recA* cells containing pRecA or pPaRecA were diluted into LB containing ampicillin (50 μg/mL). Cells were grown at 37 °C to an OD_600_ of 0.5 and treated with 1.0 μg/mL MMC. Aliquots were collected at indicated time points for immunoblotting with an anti-RecN antibody.

### Structure prediction using AlphaFold2

The structure of paRecN was predicted using AlphaFold2 implemented via ColabFold. The amino acid sequence of paRecN was used as input, and multiple sequence alignments were generated using the MMseqs2 pipeline with default parameters. Five models were generated, and the top-ranked model (ranked_0) was used for analysis.

### Fluorescence microscopy

Fluorescence microscopy analysis was carried out as described previously [24,44]. Exponentially growing cells were treated with the indicated concentrations of MMC at 37 °C, fixed with ethanol, stained with 1.0 μg/mL DAPI, and mounted on glass slides. Fluorescence microscopy was performed using a Zeiss Axioplan2 microscope. RecN foci located on or adjacent to nucleoids were classified as nucleoid-associated, whereas those at cell poles or distant from nucleoids were classified as cytoplasmic. For each strain, the localization of GFP-RecN foci was assessed in more than 100 individual cells. The results are the mean of at least five independent experiments.

### Protein purification

N-terminally hexahistidine-tagged ecRecN was purified as described previously [29]. During purification of paRecN, the N-terminally His-tagged construct (His-paRecN) failed to bind to the HisTrap HP column (Cytiva). Therefore, paRecN was expressed and purified as a C-terminally His-tagged protein (paRecN-His). The purification procedure for paRecN-His was otherwise identical to that used for ecRecN. Protein concentrations were determined using a Bio-Rad protein assay kit.

*E. coli* RecA was purified as described previously [45].

### RecA-RecN pull-down assay

RecN (2 µM) was incubated with RecA (2 µM) at 37 °C for 10 min in 10 µL of buffer A (20 mM Tris–HCl, pH 7.5, 0.01% NP-40, and 1 mM DTT) supplemented with 50 mM KCl, 2 mM MgCl₂, and 5% glycerol. The reaction mixture was then incubated with Co² ⁺ -conjugated magnetic beads (1 µL suspension), pre-equilibrated in buffer A containing 50 mM KCl, in the presence of 10 mM imidazole at 4 °C for 30 min with gentle rotation. The beads were collected and washed with buffer A containing 100 mM KCl, 2 mM MgCl₂, and 20 mM imidazole. Bound proteins were eluted with SDS sample buffer and analyzed by 10% SDS–PAGE followed by Coomassie Brilliant Blue staining.

## Supporting information

S1 Table *E. coli* strains used in this study. (PDF)

S1 Data Source data for all of the main figures. (XLSX)

S1 Fig MMC sensitivity of ∆*recN* and ∆*recA* ∆*recN* cells expressing RecA and/or RecN from the indicated species. (**A, B**) All strains were derivatives of AB1157. (**A**) ∆*recN* cells carrying plasmids expressing RecN from the indicated species. (**B**) ∆*recA* ∆*recN* cells co-expressing RecA and RecN from the indicated species. Ten-fold serial dilutions were spotted onto LB_Cm (**A**) or LB_Cm + Ap (**B**) plates containing the indicated concentrations of MMC. Three independent experiments were performed which showed similar trends.
(TIFF)

S2 Fig Isolation of paRecN gain-of-function mutants for MMC sensitivity. (**A**) MMC sensitivity of ∆*recN* cells transformed with paRecN mutant plasmids identified in the initial screen. Ten-fold serial dilutions were spotted onto LB plates in the presence or absence of MMC (0.5 μg/mL). (**B**) Amino acid sequence alignment of paRecN and ecRecN. Red boxes indicate the positions of six single-amino-acid substitutions identified in this study.
(TIFF)

S3 Fig Nucleotide sequence alignment of the coding regions of paRecN and codon-optimized paRecN (paRecN^+^). (TIFF)

S4 Fig Construction of GFP-fused RecN expression plasmid. (**A**) Schematic representation of GFP-fused RecN expression plasmids. Constructs included: GFP-RecN, in which GFP was fused directly to the N-terminus. GFP-Lk-RecN, in which GFP was fused to the N-terminus via the flexible linker. RecN-GFP, in which GFP was fused directly to the C-terminus. RecN-Lk-GFP, in which GFP was fused via the flexible linker to the C-terminus. (**B**) MMC-dependent induction of ecRecN-Lk-GFP, paRecN-Lk-GFP, or paRecN mutant Lk-GFP proteins. For comparison of expression levels, ecRecN was expressed as a C-terminal fusion construct containing the flexible linker. Protein extracts were prepared and analyzed by western blot using anti-GFP antibody. Two independent experiments were performed which showed similar trends. (**C**) MMC sensitivity of ∆*recN* cells expressing C-terminally GFP-tagged paRecN mutants (paRecN^I73T^-Lk-GFP and paRecN^R453H^-Lk-GFP). Ten-fold serial dilutions were spotted onto LB plates in the presence or absence of MMC. Three independent experiments were performed which showed similar trends.
(TIFF)

S5 Fig Purification of paRecN protein. His-ecRecN, paRecN-His, and paRecN-His mutants were purified as described under “Materials and Methods”. Purified RecN (2 µg) were analyzed on a 10% SDS gel and visualized by staining with Coomassie brilliant blue. Marker proteins (Precision plus protein prestained standard, Bio-rad) are shown on the left.
(TIFF)
