# Supplementary material for: Species-specific coevolution of RecA–RecN interfaces governs DNA double-strand break repair in Escherichia coli
Source: PLoS Genet. 2026 May 28;22(5):e1012169. doi: 10.1371/journal.pgen.1012169 (PMC13235916; doi:10.1371/journal.pgen.1012169)
Supplement: S1 Table — (PDF) [file pgen.1012169.s001.pdf]

S1 Table. *E. coli* strains used in this study

Strain	Genotype	Source
BW25113	*rph-1*, Δ*lacZ4787*, Δ*(rhaBAD)568*, Δ*(araBAD)567*, *rrnB*, *HsdR514*	NBRP
JW2669-KC	BW25113, Δ*recA*::*Km*	NBRP
JW5416-KC	BW25113, Δ*recN*::*Km*	NBRP
SN001	BW25113, Δ*recA*::*Km*, Δ*recN15002*::*Tn5*	(1)
AB1157	*thr-1*, *leuB6*, *thi-1*, *tsx-33*, *sup-37*, *supE44*, *lacY1*, *galK2*, *ara-14*, *xyl-5*, *mtl-1*, *proA2*, *his-4*, *argE3*, *rpsL31*	(2)
AMI001	AB1157, Δ(srl-*recA)306*::*Tn10*	(3)
HRS2006	AB1157, Δ*recN15002*::*Tn5*	Lab stocks
AMI002	AB1157, Δ(srl-*recA)306*::*Tn10*, Δ*recN15002*::*Tn5*	This study

Supplemental References

1. Noda, S., Akanuma, G., Keyamura, K. and Hishida, T. (2023) RecN spatially and temporally controls RecA-mediated repair of DNA double-strand breaks. *The Journal of biological chemistry*, **299**, 105466.
2. Bachmann, B.J. (1972) Pedigrees of some mutant strains of *Escherichia coli* K-12. *Bacteriol. Rev.*, **36**, 525-557.
3. Ishioka, K., Fukuoh, A., Iwasaki, H., Nakata, A. and Shinagawa, H. (1998) Abortive recombination in *Escherichia coli ruv* mutants blocks chromosome partitioning. *Genes Cells*, **3**, 209-220.
