# Supplementary figures and images for: Species-specific coevolution of RecA–RecN interfaces governs DNA double-strand break repair in Escherichia coli
Source: PLoS Genet. 2026 May 28;22(5):e1012169. doi: 10.1371/journal.pgen.1012169 (PMC13235916; doi:10.1371/journal.pgen.1012169)

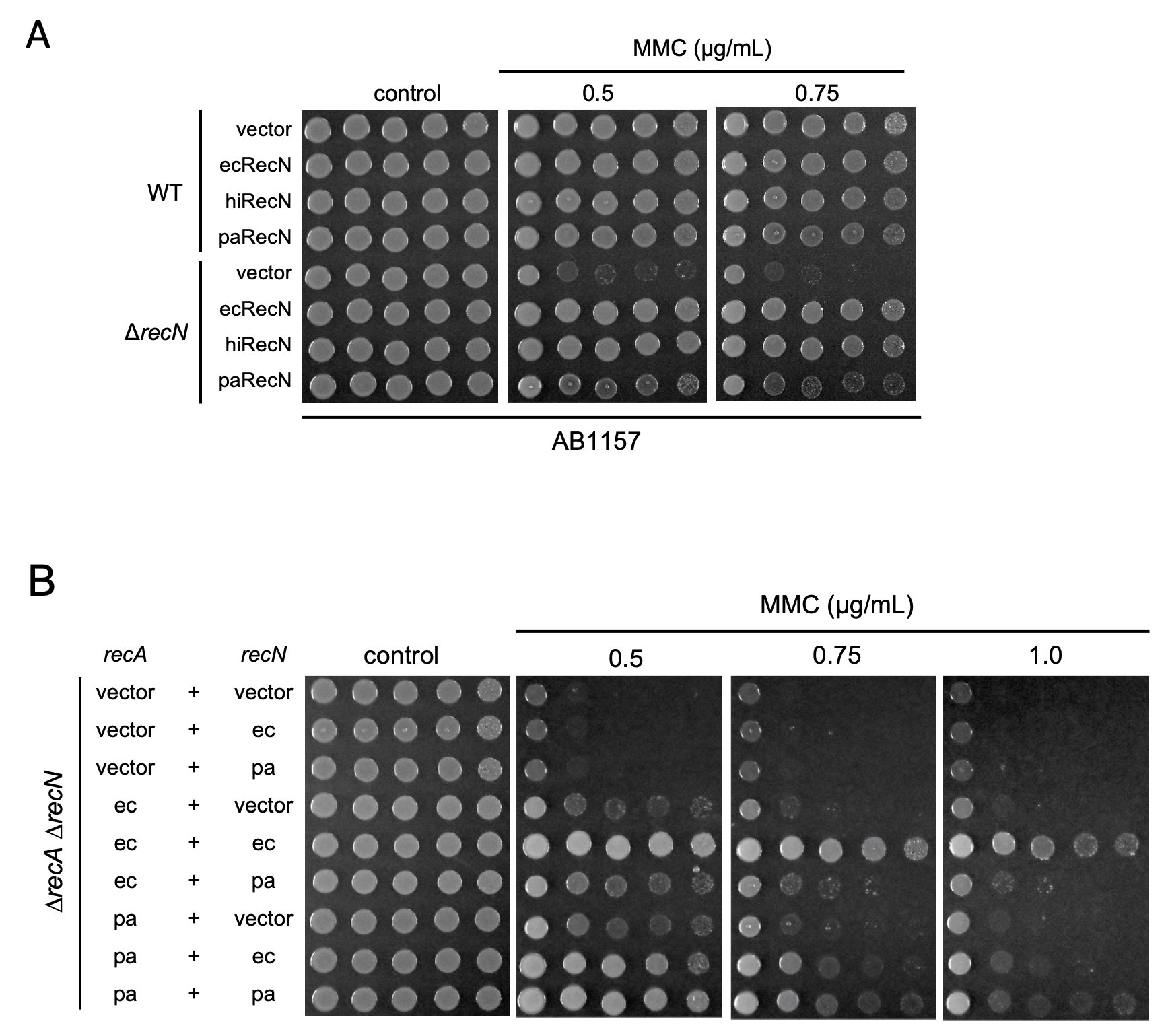

Supplement: S1 Fig — (A, B) All strains were derivatives of AB1157. (A) ∆recN cells carrying plasmids expressing RecN from the indicated species. (B) ∆recA ∆recN cells co-expressing RecA and RecN from the indicated species. Ten-fold serial dilutions were spotted onto LB_Cm (A) or LB_Cm + Ap (B) plates containing the indicated concentrations of MMC. Three independent experiments were performed which showed similar trends. (TIFF) [file pgen.1012169.s003.tiff]

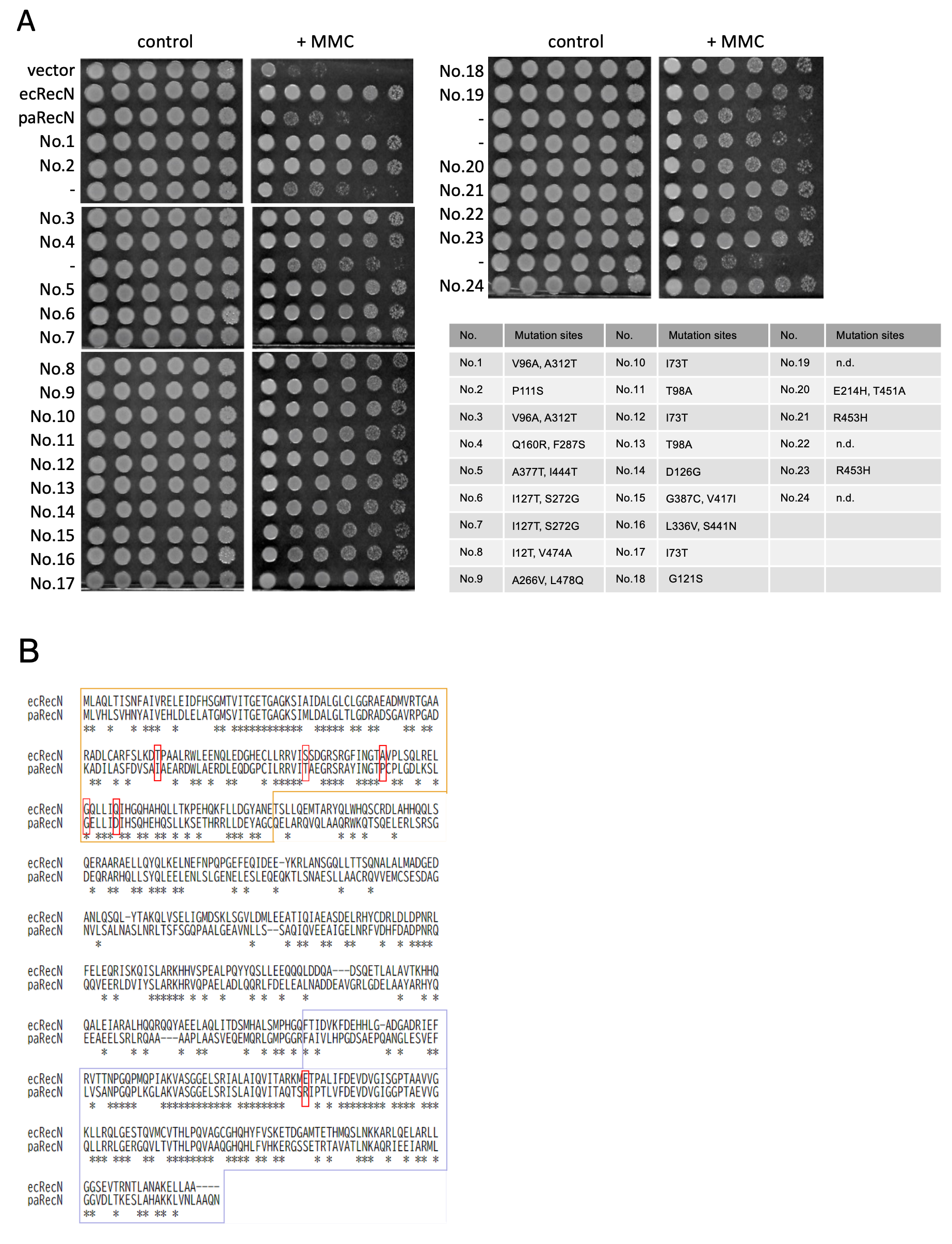

Supplement: S2 Fig — (A) MMC sensitivity of ∆recN cells transformed with paRecN mutant plasmids identified in the initial screen. Ten-fold serial dilutions were spotted onto LB plates in the presence or absence of MMC (0.5 μg/mL). (B) Amino acid sequence alignment of paRecN and ecRecN. Red boxes indicate the positions of six single-amino-acid substitutions identified in this study. (TIFF) [file pgen.1012169.s004.tiff]

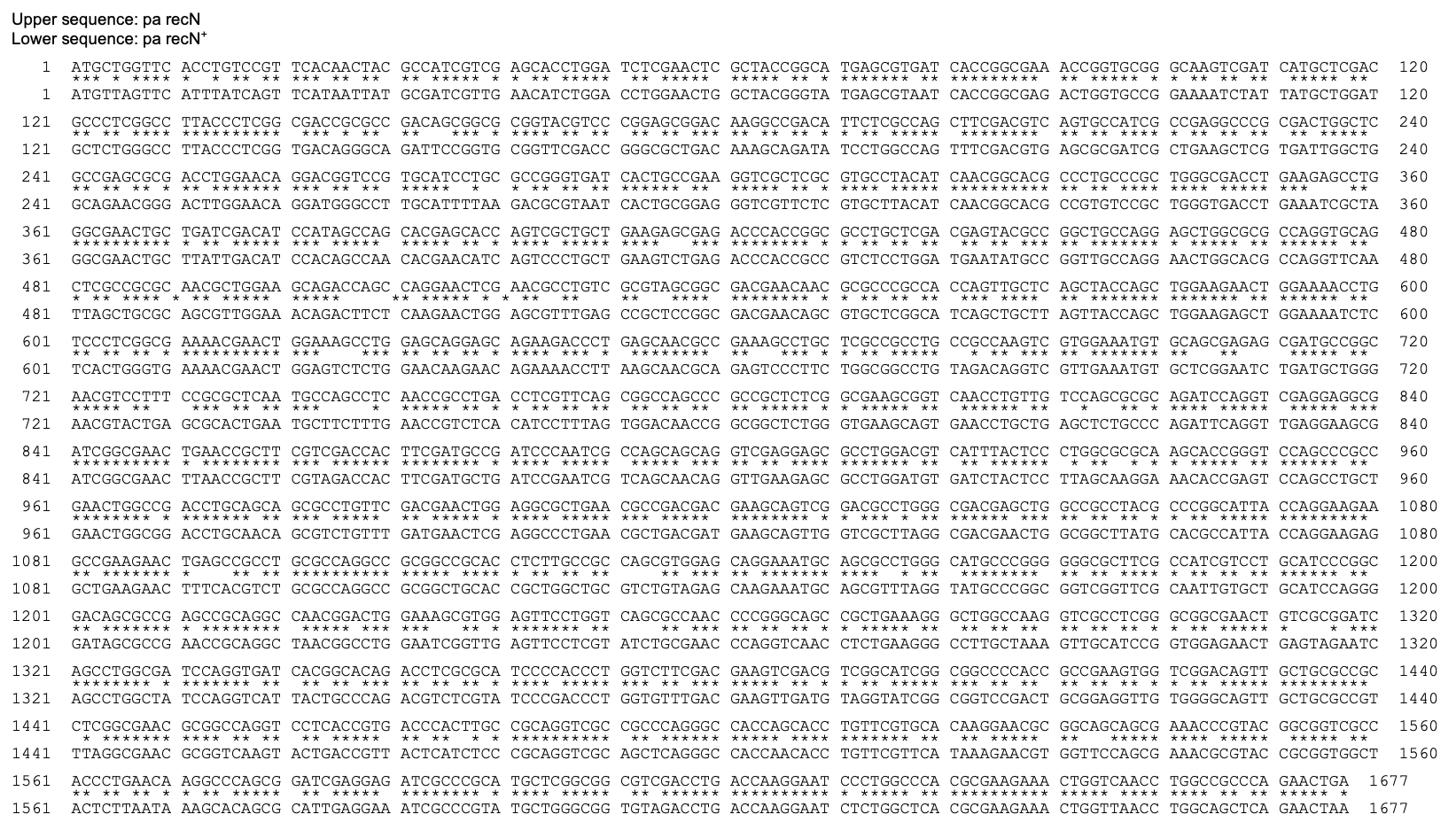

Supplement: S3 Fig — (TIFF) [file pgen.1012169.s005.tiff]

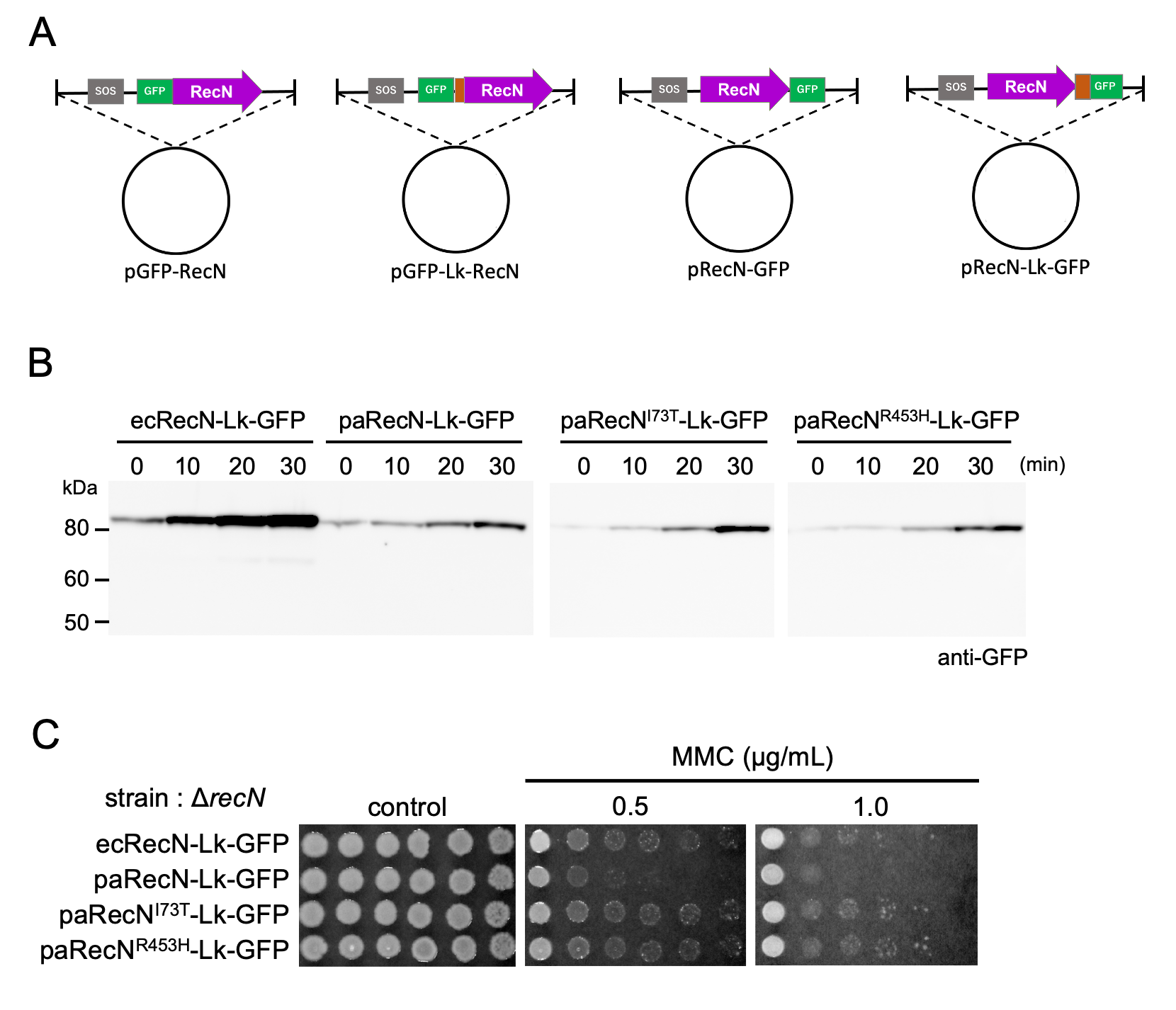

Supplement: S4 Fig — (A) Schematic representation of GFP-fused RecN expression plasmids. Constructs included: GFP-RecN, in which GFP was fused directly to the N-terminus. GFP-Lk-RecN, in which GFP was fused to the N-terminus via the flexible linker. RecN-GFP, in which GFP was fused directly to the C-terminus. RecN-Lk-GFP, in which GFP was fused via the flexible linker to the C-terminus. (B) MMC-dependent induction of ecRecN-Lk-GFP, paRecN-Lk-GFP, or paRecN mutant Lk-GFP proteins. For comparison of expression levels, ecRecN was expressed as a C-terminal fusion construct containing the flexible linker. Protein extracts were prepared and analyzed by western blot using anti-GFP antibody. Two independent experiments were performed which showed similar trends. (C) MMC sensitivity of ∆recN cells expressing C-terminally GFP-tagged paRecN mutants (paRecNI73T-Lk-GFP and paRecNR453H-Lk-GFP). Ten-fold serial dilutions were spotted onto LB plates in the presence or absence of MMC. Three independent experiments were performed which showed similar trends. (TIFF) [file pgen.1012169.s006.tiff]

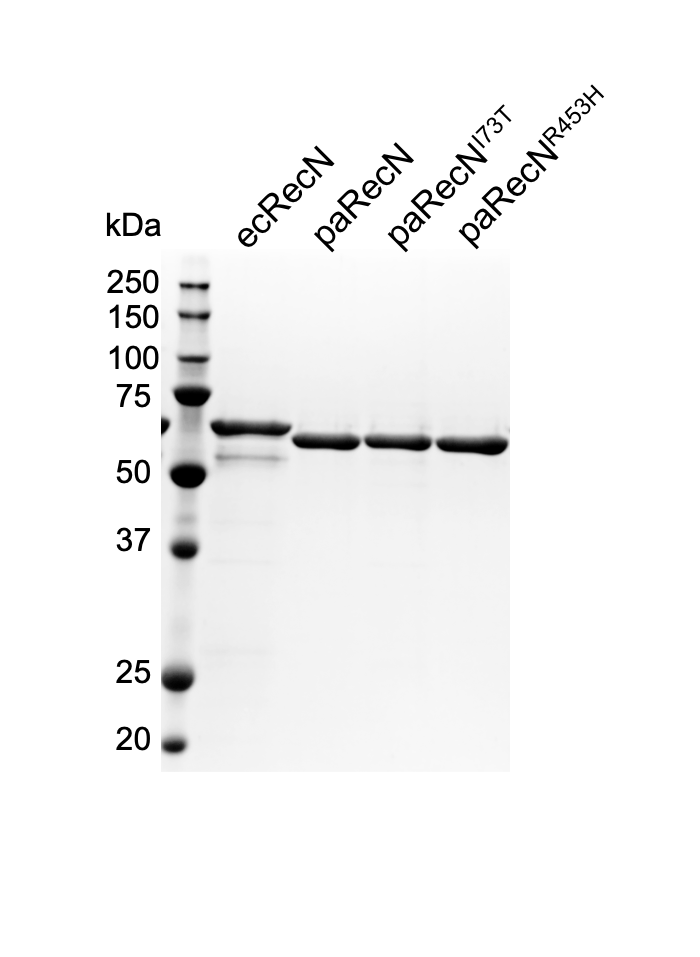

Supplement: S5 Fig — His-ecRecN, paRecN-His, and paRecN-His mutants were purified as described under “Materials and Methods”. Purified RecN (2 µg) were analyzed on a 10% SDS gel and visualized by staining with Coomassie brilliant blue. Marker proteins (Precision plus protein prestained standard, Bio-rad) are shown on the left. (TIFF) [file pgen.1012169.s007.tiff]
